# Complexities of Assessing the Disease Burden Attributable to Leishmaniasis

**DOI:** 10.1371/journal.pntd.0000313

**Published:** 2008-10-29

**Authors:** Caryn Bern, James H. Maguire, Jorge Alvar

**Affiliations:** 1 Division of Parasitic Diseases, National Center for Zoonotic, Vector-Borne and Enteric Diseases, Centers for Disease Control and Prevention, Atlanta, Georgia, United States of America; 2 Harvard Medical School and Division of Infectious Disease, Brigham and Women's Hospital, Boston, Massachusetts, United States of America; 3 Department for the Control of Neglected Tropical Diseases (HTM/NTD/IDM), Leishmaniasis Control Program, World Health Organization, Geneva, Switzerland; London School of Hygiene & Tropical Medicine, United Kingdom

## Abstract

Among parasitic diseases, morbidity and mortality caused by leishmaniasis are surpassed only by malaria and lymphatic filariasis. However, estimation of the leishmaniasis disease burden is challenging, due to clinical and epidemiological diversity, marked geographic clustering, and lack of reliable data on incidence, duration, and impact of the various disease syndromes. Non-health effects such as impoverishment, disfigurement, and stigma add to the burden, and introduce further complexities. Leishmaniasis occurs globally, but has disproportionate impact in the Horn of Africa, South Asia and Brazil (for visceral leishmaniasis), and Latin America, Central Asia, and southwestern Asia (for cutaneous leishmaniasis). Disease characteristics and challenges for control are reviewed for each of these foci. We recommend review of reliable secondary data sources and collection of baseline active survey data to improve current disease burden estimates, plus the improvement or establishment of effective surveillance systems to monitor the impact of control efforts.

## Introduction

Leishmaniasis comprises a complex of vector-borne diseases, caused by more than 20 species of the protozoan genus *Leishmania*, and ranging from localized skin ulcers to lethal systemic disease [1],[2]. Leishmaniasis is classified as one of the “most neglected diseases” [3], based on the limited resources invested in diagnosis, treatment, and control, and its strong association with poverty [4]. Published disease burden estimates place leishmaniasis second in mortality and fourth in morbidity among all tropical diseases [5].

The tools exist to achieve much better control of leishmaniasis. Research efforts over the past decade have augmented the range of field-applicable diagnostic tools and effective antileishmanial drugs available, especially for visceral leishmaniasis [6]. Appropriate use of vector control interventions, such as insecticide-treated nets and indoor residual spraying, would greatly reduce incidence [7]–[10]. Nevertheless, leishmaniasis control efforts have been impeded by the lack of a simple strategy, such as a vaccine. With the impetus of a “rapid impact package” for the small group of neglected diseases amenable to control through mass drug administration [11], leishmaniasis is in danger of becoming an even more neglected disease.

The characteristics that complicate large-scale interventions against leishmaniasis also present a challenge to preparing realistic disease burden estimates. Leishmaniasis incidence is geographically heterogeneous: while the rate across a region may appear low, focal areas are intensely affected [9],[12]. Leishmaniasis causes highly varied clinical syndromes, each presenting distinct diagnostic challenges, most requiring prolonged, expensive drug therapy, and each contributing differently to disease burden. Interactions with malnutrition and HIV alter the clinical course, and complicate therapeutic strategies [13],[14]. Epidemiologic features and the choice of appropriate control measures vary with parasite species and geographic area, depending on reservoir hosts and biological aspects of the vectors [2]. Finally, the impact on patients and their families does not end with health effects, but includes the social and psychological stigma of visible lesions and disfigurement, and significant economic losses [4]. This article reviews the major clinical features and epidemiology of leishmaniasis, and describes aspects of the disease burden in the most important foci.

## Clinical Features

The most common syndrome is localized cutaneous leishmaniasis (CL), most frequently caused by *Leishmania major* and *L. tropica* in the Old World, and *L. braziliensis*, *L. mexicana*, and related species in the New World [1],[15]. Spontaneous healing is the rule, but requires months to years, and varies by species [15]. Mucosal leishmaniasis (ML) usually occurs months or years after healing of primary CL, most commonly due to *L. braziliensis*, and can cause destruction of the nasal septum, palate, and other mucosal structures, leading to devastating facial mutilation and, rarely, death from airway involvement [16]. Other complicated forms include disseminated cutaneous leishmaniasis (DCL), diffuse nodular non-ulcerating disease, and leishmaniasis recidivans, localized slowly progressive non-healing lesions. Both are rare, difficult to treat, and can be severe.

Visceral leishmaniasis (VL) is usually caused by *L. donovani* and *L. infantum*, and is characterized by progressive fever, weight loss, splenomegaly, hepatomegaly, hypergammaglobulinemia, and pancytopenia [1]. Complications include immunosuppression and secondary bacterial infections, hemorrhage, anemia, and, when kala-azar occurs during pregnancy, fetal wastage or congenital leishmaniasis [17]. Kala-azar is lethal in nearly all untreated cases [18],[19]. Even in treated patients, case-fatality rates are often 10% or higher; jaundice, wasting, severe anemia, and HIV co-infection are associated with increased risk of mortality [9],[20],[21].

Post-kala-azar dermal leishmaniasis (PKDL) is a chronic rash seen in apparently cured kala-azar patients in South Asia and the Horn of Africa [22],[23]. PKDL patients present with erythematous or hypopigmented macules, sometimes progressing to plaques or nodules. In Sudan, PKDL is reported to resolve without treatment in most mild cases, while the condition is said to require universal treatment in South Asia [23]. However, recent data from Bangladesh suggest that a proportion of PKDL cases self-resolve in South Asia as well [24]. Up to 60% of kala-azar patients develop PKDL in Sudan [23]; in South Asia, prospective data are lacking, but the cumulative incidence is currently thought to be in the range of 10%–20% [22],[24].

HIV–leishmaniasis co-infection poses a growing problem in developing countries [13]. In HIV-infected individuals without severe immunosuppression, manifestations are similar to those in immunocompetent persons. Among those with CD4+ T lymphocyte counts <200 cells/µL, manifestations of leishmaniasis may be more severe or affect unusual sites such as the gastrointestinal tract [25]. In the absence of highly active antiretroviral therapy (HAART), the relapse rate after treatment approaches 100% [13].

## Epidemiology and Ecology

The leishmaniases are transmitted to humans in sylvatic, domestic, and peridomestic cycles ranging from cities to deserts and rain forests on every continent except Australia and Antarctica (Table 1). Nevertheless, the human disease burden is relatively concentrated; 90% of VL cases occur in India, Bangladesh, Nepal, Sudan, Ethiopia, and Brazil, while 90% of CL occurs in Afghanistan, Algeria, Iran, Saudi Arabia, Syria, Brazil, Colombia, Peru, and Bolivia [26],[27]. The distribution is dynamic: Colombia and Ethiopia have recently joined this list, and Pakistan currently faces a large epidemic of CL in Baluchistan and Sindh (World Health Organization [WHO], unpublished data). Climate change and other environmental changes have the potential to expand the geographic range of the vectors and leishmaniasis transmission in the future [28].

**Table 1:** Characteristics of the major geographic foci of leishmaniasis.

Major Geographic Focus	Predominant Clinical Forms	Species	Setting	Major Reservoir Hosts	Epidemiologic Pattern
South Asia (India, Bangladesh, Nepal)	VL, PKDL	*L. donovani*	Domestic, peridomestic; agricultural villages	Human	Endemic with superimposed outbreaks
East Africa (Sudan, Ethiopia, Kenya, Uganda, Somalia)	VL, PKDL	*L. donovani*, *L. infantum*	Sylvatic, peridomestic	Humans, dogs (?), sylvatic mammals	Endemic with superimposed outbreaks
Mediterranean basin, Middle East, western Asia to China	VL	*L. infantum*	Domestic, peridomestic	Domestic dogs	Endemic
Brazil, sporadic elsewhere in Latin America	VL	*L. infantum*	Domestic, peridomestic, including periurban	Domestic dogs	Endemic with periodic outbreaks
Middle East to Afghanistan, parts of North and East Africa	Anthroponotic CL	*L. tropica*	Urban domestic, peridomestic	Human, hyraxes, dogs (?)	Endemic with superimposed outbreaks
Northern Africa, Western and Central Asia	Zoonotic CL	*L. major*	Sylvatic, encroachment on peridomicile	Sylvatic rodents	Sporadic human cases, periodic outbreaks
East African highlands (Ethiopia, Kenya)	Zoonotic CL	*L. aethiopica*	Sylvatic, peridomestic	Hyraxes	Endemic in communities near hyrax habitat
Peru and Ecuador	CL (uta)	*L. peruviana*	Andean valleys	Wild rodents, dogs (?)	Endemic
Northern South America	CL (pian-bois)	*L. guyanensis*	Tropical forests	Sloths, anteaters, oppossum	Sporadic human cases related to forest activities
Central and South America (Honduras to Ecuador)	CL and ML	*L. panamensis*	Tropical forest and deforested areas	Sloths	Sporadic human cases related to forest activities
Latin America (Mexico to Argentina)	CL and ML	*L. braziliensis*	Tropical forest and deforested areas	Not proven, but found in sloths, oppossums; dogs may be domestic reservoir	Human cases related to forest activities; also domestic, peridomestic in recent years
Americas (southern Texas to Bolivia)	CL	*L. mexicana*	Varied sylvatic settings (humid forests to dry scrub)	Variety of forest rodents and marsupials	Sporadic human cases related to forest activities

In sylvatic cycles, such as those in New World rain forests and the deserts of Central Asia, animal reservoir hosts can maintain enzootic transmission indefinitely without human disease. Sporadic or epidemic leishmaniasis occurs when humans enter the sylvatic habitat for economic or military purposes, or when human habitation encroaches on the sylvatic setting. In domestic cycles, humans or dogs form the predominant or sole infection reservoir. The foci that account for the largest number of human cases, for example, VL in South Asia and CL in Afghanistan, usually reflect anthroponotic transmission [1],[29]. In anthroponotic VL foci, the reservoir includes humans with untreated kala-azar [9], but PKDL patients may maintain the infection between kala-azar epidemics [30]. Up to half the population in highly affected foci may have asymptomatic leishmanial infection; the contribution of such individuals to transmission is presumed to be less than for active kala-azar, but has never been quantified [31],[32].

## Disease Burden Estimates

The most objective measures of disease burden are incidence, prevalence, and mortality. Several derived measures incorporate indicators of disease severity, disability, and/or quality of life into composite outcomes that can be compared across diseases [33]. Currently, the most widely used measure is “disability-adjusted life years lost” (DALY) [5],[33]. Leishmaniasis DALY estimates are based on (1) figures assumed for regional incidence and prevalence, (2) assumed case-fatality rates, and (3) assigned disability weights for CL and VL [33]. For leishmaniasis, there are major uncertainties and sparse documentation for the assumptions underlying all three of these components. The empirical basis and derivation of global and regional leishmaniasis incidence and prevalence figures have not been documented since 1991 [34],[35]. Passive surveillance is generally given as their basis, but leishmaniasis is notifiable in only 33 of 88 endemic countries [26]. Substantial underreporting is widely acknowledged [26], but its magnitude has rarely been measured, and in studies, has varied from 2-fold to 40-fold [36]–[38]. In many countries, the majority of leishmaniasis cases are treated by non-governmental organizations or in the private sector, but these cases are not usually included in surveillance data, exacerbating underreporting. Underreporting is likely to vary greatly, not only among countries and depending on the clinical syndrome, but even between localities in the same district, based on distance to health care, availability of private providers and of antileishmanial drugs, the presence of research groups, and local awareness of the disease. Differential underreporting is labor-intensive to document, and precludes valid generalization of incidence and reporting rates presented in research studies. Underreporting of deaths is even more pronounced. One study from Sudan estimates that 91% of all kala-azar deaths went unrecognized [39], while data from a village-based study in India suggest that as many as 20% of VL patients, disproportionately poor and female, died before their disease was recognized [40]. The origin and derivation of the disability weights assigned for VL and CL are not documented [33]. In the absence of data to address these shortcomings, this article will describe the characteristics of leishmaniasis that contribute to the disease burden in the most important foci, and aspects that should be taken into account in future attempts to quantify its impact and monitor control programs.

## Horn of Africa

In global estimates, the Horn of Africa (Sudan, Ethiopia, Kenya, Somalia) accounts for the second largest number of annual VL cases, after South Asia [41]. Transmission dynamics are complex, involving parasites identified by standard laboratory techniques as both *L. donovani* and *L. infantum* [42],[43], and two distinct ecological settings, semi-arid regions in the north where *Phlebotomus orientalis* is the major vector, and the savanna and forest areas in the south where *P. martini* and *P. celiae* are found in association with *Macrotermes* termite mounds [44],[45]. Investigators have suggested that VL originated in the Sudan, based on the ancestral position of the circulating parasites in genetic analyses [46],[47]. While sporadic sylvatic VL transmission is well recognized [48], sustained peridomestic and domestic cycles in villages, and explosive epidemics affecting populations displaced in recent wars, account for the bulk of human cases [42],[43],[49]. In zoonotic foci, both sylvatic rodents [50] and domestic dogs [51] may act as infection reservoirs, but large outbreaks are usually thought to involve anthroponotic transmission.

The association of leishmaniasis epidemics with war, ecological disasters, famine, and forced migration is most marked in the Horn of Africa. During the long civil war in Sudan, hundreds of thousands of VL cases occurred, causing the deaths of 30%–60% of the population in many communities [49]. The high attack rates among all ages and very high mortality rates were due to the confluence of displaced populations with no immunity, high rates of malnutrition, and lack of treatment access [20],[49],[52],[53]. Malnutrition is a major determinant of both progression to and severity of clinically manifest VL [14],[54],[55], and greatly increases the case-fatality rate [20]. Poverty, poor housing, crowded conditions, lack of personal protective modalities such as bed nets, environmental degradation, and collapse of health care systems intensify the spread and impact of VL in these settings. Seasonal labor movements may also spread the disease: introduction of the parasite by returning migrant farm workers is thought to have initiated an outbreak of VL in north central Ethiopia, an area not previously endemic [42]. VL epidemics with similar underlying causes have occurred recently in Somalia [56],[57] and Kenya [58],[59]. Cutaneous leishmaniasis may also accompany population displacements, for example, in Kurdish refugee camps in north Syria, Sudanese refugee camps in Chad, and among returned refugees in Afghanistan [60]–[62] (and WHO, unpublished data).

HIV–VL co-infection further complicates the picture by accelerating the progression of both diseases and making VL virtually untreatable in the absence of HAART [13],[63]. The Tigray region, bordering Eritrea and Sudan, has high rates of both HIV and VL among the many soldiers and seasonal workers; seasonal workers often sleep under *Acacia* trees where sand flies rest, increasing risk of VL [44]. In recent studies in Tigray, 20%–30% of VL patients were co-infected with HIV; co-infection was associated with a 5-fold increase in mortality within 6 months, lower clinical cure rates with frequent relapses, and increased side effects from antimonial drugs [64],[65]. HIV–VL co-infected patients are highly infectious to sand flies [66], and have the potential to spread resistant parasite clones, posing an additional threat to control programs.

## South Asia (India, Nepal, and Bangladesh)

If Sudan is the original home of VL, South Asia is its domestic heartland. In the 19th century, devastating outbreaks of a chronic progressive febrile illness with cachexia, hepatosplenomegaly, and high fatality rates were reported in Bengal and Assam, and retrospectively thought to be the first recorded VL epidemics [19]. In 1903, Leishman and Donovan first described the organism that now bears their names in patients infected in India [67],[68]. Today, South Asia is estimated to account for 60% of the global VL disease burden [41], with a sustained endemic focus stretching from Bihar and Bengal in northeastern India, across the border into southeastern Nepal, and to the east into central and western Bangladesh. The parasite in South Asia is transmitted by *Phlebotomus argentipes*, an endophilic vector that rests in human and animal dwellings in densely populated agricultural villages. Kala-azar incidence fell substantially during the indoor residual insecticide spray campaigns of the malaria eradication effort of the 1950s and 1960s, but the disease returned in the 1970s and transmission has been sustained since then [69],[70].

Superimposed on this poorly controlled endemic picture, India has experienced recurrent epidemics in the 1970s and early 1990s, and Bangladesh has seen a progressive increase in VL incidence from the mid-1990s to the present that shows no signs of abating [12],[69],[70]. In most areas, there is a fairly stable incidence of two to three kala-azar cases per 1,000 population per year [36], but with localized foci of intense transmission and 10-fold higher annual incidence rates [71]. Transmission hot spots may be sustained for several years, but then appear to burn out, limited by saturation of the susceptible population [9]; increases in incidence are then seen in neighboring areas. Several years after peaks in kala-azar incidence, the same communities may see large numbers of PKDL cases, in an echo of the original kala-azar outbreak [24]. PKDL patients remain infectious for years to decades [30], and require prolonged antileishmanial treatment, up to 120 days [72], representing a significant challenge to health care systems in which kala-azar patients experience difficulty obtaining much shorter treatment courses [73].

Facility-based studies from South Asia often report higher kala-azar incidence in males than females [74]; community-based data suggest that there is little difference in incidence by sex, but substantial differences in care-seeking behavior [40],[71]. In South Asia, the mean duration of kala-azar illness before treatment is 3–5 months; on average, women are ill longer than men, and are more likely to die from the disease [8],[40],[71],[75],[76]. In one highly affected village in Bangladesh, reproductive-age women were three times as likely to die from kala-azar compared to men or children; kala-azar accounted for 23% of all deaths, and 80% of those in adult women [71],[73]. Qualitative data from the same village suggest that women experience higher barriers to seeking care [71],[75]; poorer baseline iron, zinc, and vitamin A status may also play a role in higher morbidity and mortality among women [31].

Although the morbidity and mortality caused by kala-azar and PKDL are substantial, the impact on affected individuals and their families is compounded by the expense and time involved in gaining access to appropriate diagnosis and treatment. The cost of caring for a patient with kala-azar in South Asia (US$80–US$120) approaches or surpasses the annual per capita income, and substantial additional income is lost by patients and family members unable to work [73], [77]–[79]. In Bihar and southern Nepal, costs have been multiplied many-fold by resistance to antimonial drugs and the imperative to use more expensive alternatives [6],[80],[81]. The upsurge in PKDL cases now seen in Bangladesh will also increase difficulties for patients and their families; even if the drug is supplied gratis, the 120-day parenteral treatment course entails many other costs, such as payments for daily injections and transport to the health care facility, and is associated with much lost work time. Anecdotally, a number of PKDL patients died suddenly during treatment, consistent with antimonial cardiotoxicity [24].

## Visceral Leishmaniasis in Brazil and Other Parts of Latin America

From Mexico to Argentina, *L. infantum* (synonym *L. chagasi*) is transmitted from dogs to humans primarily by *Lutzomyia longipalpis*, a vector well adapted to the domestic and peridomestic environment. Brazil accounts for 90% of reported VL cases in the Americas, and is the third most important VL focus globally [82]; unlike other major VL foci, case reporting is mandatory in Brazil, and surveillance data are more complete. However, American VL is in the midst of dramatic changes in transmission patterns and a marked geographic expansion, superseding previous estimates of disease burden.

VL traditionally occurred in poor rural areas in dry northeast Brazil. In the early 1980s, the first of a series of urban epidemics occurred in Teresina [83], followed by outbreaks in São Luis, Natal, Fortaleza, and elsewhere [84]. Periurban VL outbreaks followed the massive migration of rural populations to the periphery of large cities because of drought, loss of farmland, and poverty [85]. In rapidly growing, densely settled favelas, environmental degradation, precarious living conditions, inadequate sewage and garbage disposal, and close contact with dogs and other domestic animals promote vector proliferation and disease transmission [86],[87]. The reported VL incidence in Brazil doubled from a mean of 1,500 cases per year in the 1980s to more than 3,000 per year from 2000 to 2005; the disease now occurs in urban, periurban, and rural areas as far south as the states of São Paulo and Mato Grosso do Sul, in addition to the traditionally endemic northeast [82]. Children, particularly those with malnutrition, have the highest risk for kala-azar [14],[55]; the case-fatality rate approaches 10% in some centers, despite good availability of treatment [88]. Although the geographic ranges of HIV infection and VL overlap and continue to expand, the number of reported cases of VL with AIDS (176 from 2001–2005) has been fewer than expected, perhaps because of free, universal distribution of antiretroviral drugs by the Brazilian government, and some degree of underrecognition by clinicians [89]. An additional 315 cases of VL–HIV co-infection without AIDS are estimated to have occurred during that period [90].

Control of zoonotic VL has proved difficult. From 1999–2005, human VL incidence remained high despite aggressive control efforts centered on culling of infected dogs and insecticide application [91]. The failure of the previous control strategy [92], and demonstration of canine infections and subclinical human infections in areas not previously endemic [93], have led to a revised national strategy that includes surveillance and preventive measures in areas considered at risk for infection, even in the absence of clinical cases [82].

## Cutaneous Leishmaniasis in the Americas

At least 12 different *Leishmania* species cause American CL, and the disease occurs in every country from the United States to Argentina, except Uruguay and Chile. Until recently, Brazil and Peru reported the first and second highest incidence in the Americas [15],[26]. However, with more than 15,000 reported cases in 2005 and 2006, Colombia now ranks second after Brazil (>30,000 per year); thousands of cases occur in Peru (∼6,500 per year) and elsewhere in Latin America as well [15],[89],[94],[95]. The epidemiology of CL in the Americas is complex, with intra- and inter-specific variation in transmission cycles, reservoir hosts, sand fly vectors, clinical manifestations, and response to therapy [96]. Studies often demonstrate five or more species causing lesions in the same area [97]–[99].

There has been an expansion both in the geographic range and risk factors for CL transmission. In the past, American CL was predominantly an occupational disease, related to activities in forests and other enzootic areas. Occupational exposures remain important, as demonstrated by 3,163 reported CL cases among Colombian soldiers infected during patrols in forested areas held by insurgents in 2004 [60]. However, widespread deforestation has led to a rapid increase in cases, rather than a decrease as once predicted, and to peridomestic, periurban, and even urban transmission [100]. For example, from 1980 to 2001, there was a 10-fold increase in CL incidence and spread to all of the states of Brazil [63],[101]. Outbreaks in newly arrived, immunologically naive immigrants have occurred in new settlements in previously forested areas [102] and among economic migrants in the lowlands of Bolivia [103]. A contrasting report documents new occurrence of *L. amazonensis* infection in a settled population in a sub-Andean region of Bolivia at 1,450–2,100 meters above sea level, presumably reflecting spread from its traditional lowland focus [104].

CL patients face social stigma and isolation [27]. A study from Colombia reported that cutaneous ulcers in a woman can be the pretext for spousal abandonment [105]. Among Ecuadorian villagers, almost 70% of persons believed that CL interfered with the capacity to work, while 82% stated that the presence of an ulcer or scar diminished self-esteem [106]. Participants mentioned over 150 different treatments for CL, including potentially harmful application of acids, gasoline, and lighted matches [106]. Disfiguring, mutilating, and occasionally life-threatening lesions of ML have been reported in 25%, 14%, 2%, and 0.3% of persons with *L. braziliensis* infections in Bolivia, Peru, Colombia, and Venezuela, respectively [107]. Diffuse cutaneous leishmaniasis, like ML, does not heal spontaneously and is difficult to treat; although rare, this syndrome can occur in persons with *L. amazonensis* or *L. mexicana* infections, or co-infection with HIV and other species of *Leishmania* [101]. The nodular lesions resemble those of lepromatous leprosy, and persons with DCL can suffer stigma similar to that associated with leprosy.

CL in the Americas is a disease of the poor; in many countries, patients and their families shoulder the high cost of treatment, and suffer substantial lost income. For example, in Guatemala, the cost of treatment is about US$250, beyond the means of most rural inhabitants [27]. The disease also causes a major financial burden on public health systems. Treatment is provided free of charge by the governments of Colombia, where the cost of pentavalent antimony is approximately US$345 per person cured [108], and in Brazil, which has spent the equivalent of US$2.5 million to treat 35,000 persons with antimonial drugs, and an additional US$500,000 to treat 95 persons with liposomal amphotericin [27].

## Anthroponotic CL in Afghanistan and Southwestern Asia

Of the major forms of leishmaniasis, the only historically urbanized form is anthroponotic CL due to *L. tropica*, as illustrated by its vernacular names, “Baghdad boil”, “Aleppo boil”, “Balkh sore”, and others [46]. Although infections in dogs and other animals have been documented, the disease is characterized by large outbreaks in densely populated cities, especially in the setting of war and large-scale population migration. In Syria, especially the traditional focus in the city of Aleppo, a marked increase to more than 15,000 cases per year was documented during the 1990s, with only a temporary decline when insecticide spray programs were instituted in 1991 [109]. A huge CL epidemic has occurred in Afghanistan since 1992, with estimates of 200,000 cases in Kabul alone [110]. The annual CL incidence in Kabul peaked at 12% in 1996, and averaged 3% per year from 1992 to 2002 [29],[62]. The association between migration and CL transmission may be more complex than originally postulated: while migrants within Kabul were at the highest risk of CL, possibly because of economic disadvantage, immigrants from outside Kabul were at no higher risk, but appeared to fuel local transmission by adding to the pool of susceptible residents [62]. Transmission occurred within the household, even up to second floor apartments [111], and often resulted in facial lesions, especially in women and children [62]. Women with lesions were considered unfit to marry, have children, or breastfeed, and children with lesions were sometimes ostracized by playmates [112].

## Conclusions and Recommendations

Current methods of assessing disease burden fail to take into account the clinical and epidemiological diversity of leishmaniasis, and the intense medical, social, and economic impact within highly affected foci. Furthermore, existing passive surveillance data are grossly inadequate to be used to make reliable estimates. Active, rigorous assessments of the true incidence, morbidity, mortality, current transmission patterns, and non-health effects of leishmaniasis are urgently needed. The following steps are recommended to achieve better estimates of current disease burden, and establish systems to monitor the impact of control measures:

1. Assessment of the critical needs for data by geographic focus, and development of focus-specific strategies for filling in the many data gaps. Leishmaniasis foci targeted for elimination or intensified control should be given highest priority. In areas without reliable data, on-the-ground rapid epidemiologic assessments may be necessary to develop an appropriate plan for data collection. Strategies include reviews of existing data, baseline field surveys, and establishment of surveillance systems. More than one technique may be needed in a single focus.
2. Reviews of reported incidence and mortality data. Potential data sources include existing surveillance systems, hospital and specialized treatment facility records, and past surveys. Data analysis should be preceded by a critical assessment of data sources, quality, and potential biases.
3. Where indicated, baseline surveys to collect empirical incidence and prevalence data, with the focus on the most affected regions, and those with planned elimination or intensified control programs. Appropriate case definitions and techniques to capture illness onset, severity, and duration should be developed and tested before deployment. Surveys must be carefully designed to avoid biases due to disease clustering, employ valid statistical methods, ensure adequate sample size, and, if possible, employ new mapping technologies. Specialized sampling and analysis methods, such as adaptive sampling [113], may be necessary to address the clustered transmission pattern of leishmaniasis. Wherever practical, risk factor assessments should be incorporated into baseline surveys in order to guide control efforts and make the best use of limited resources.
4. The data collected in rapid assessments and baseline surveys should be used to evaluate and improve existing leishmaniasis surveillance and reporting systems. In areas without existing surveillance, systems should be established. Where complete coverage is not feasible, sentinel surveillance based on carefully selected, sustainable sites with known catchment populations may be the best option to provide data to evaluate trends over time [114]. Systems should be tailored to local conditions, but should be subject to ongoing evaluation to ensure reliability and appropriateness to monitor the impact of control programs.
5. Studies and/or surveillance system components should be designed to provide measures of disease impact, severity, and duration that realistically reflect the heterogeneity of leishmaniasis. This effort should incorporate new thinking on disease burden assessment [115], for example, including the impact of non-health outcomes, such as economic costs, and interaction with malnutrition or other conditions.
6. The data resulting from the above efforts should be incorporated into a more precise assessment of disease burden, including periodic reassessments as conditions change and new assessment tools become available.
